# ERRATUM: Characterization of βlactoglobulin and κcasein genotypes PCR-RFLP in dairy cattle from Panama

**DOI:** 10.29374/2527-2179.bjvm013626

**Published:** 2026-09-15

Due to authors honest mistake the article “Characterization of βlactoglobulin and κcasein genotypes PCR-RFLP in dairy cattle from Panama” (https://doi.org/10.29374/2527-2179.bjvm001026), published in Brazilian Journal of Veterinary Medicine, 48, e001026, 2026, was published with some errors along the text.

On page 1, both authors Anabel Argelis García and Jenyfer Pérez Garcíathey share first authorship.

Where it reads:

Anabel Argelis García^1,2^, Jenyfer Pérez García^1†^, Dilcia Sambrano^2^ , Fermín Acosta^2,3^, César Edwards^4^, José Torres^4^, Diomedes Trejos^4^, Amador Goodridge^2,3*^ & Cecilia Escobar^5*^

It should read:

Anabel Argelis García^1,2^**^†^**, Jenyfer Pérez García^1^**^†^**, Dilcia Sambrano^2^ , Fermín Acosta^2,3^, César Edwards^4^, José Torres^4^, Diomedes Trejos^4^, Amador Goodridge^2,3*^ & Cecilia Escobar^5*^

On page 1, both authors Amador Goodridge and Cecilia Escobar should be corresponding authors.

Where it reads:

*Correspondence**Amador Goodridge**Instituto de Investigaciones Científicas y Servicios de Alta Tecnología – INDICASAT AIP Clayton, Building 208 CEP 0843-01103 - Ciudad del Saber (Panama City), Panama E-mail: agoodridge@indicasat.org.pa

It should read:

*Correspondence**Amador Goodridge / Cecilia Escobar**Instituto de Investigaciones Científicas y Servicios de Alta Tecnología – INDICASAT AIP Clayton, Building 208 CEP 0843-01103 - Ciudad del Saber (Panama City), Panama E-mail: agoodridge@indicasat.org.pa

Ministerio de Desarrollo Agropecuario (MIDA), Curundu, Panama, Panama

ceciligdeescobar@hotmail.com

In the running title on all pages, where it reads:

Characterization of **βlactoglobulin** and **κcasein** genotypes PCR-RFLP in dairy cattle from Panama

It should read:

Characterization of **β-lactoglobulin** and **κ-casein** genotypes PCR-RFLP in dairy cattle from Panama

On pages 1, 3 and 10, where it reads:

**βlactoglobulin**

It should read:

**β-lactoglobulin**

On pages 1 and 10, where it reads:

**κcasein**

It should read:

**κ-casein**

On pages 1, 2, 3, 4, 5 and 6, where it reads:

**βLg**

It should read:

**β-Lg**

On pages 1, 2 and 6, where it reads:

**PCRRFLP**

It should read:

**PCR‑RFLP**

On pages 1 and 2, where it reads:

**populationlevel**

It should read:

**population-level**

On pages 1 and 6, where it reads:

**populationbased**

It should read:

**population-based**

On page 3, where it reads:

**NgöbeBuglé**

It should read:

**Ngöbe-Buglé**

On page 3, where it reads:

**nonrandomized, conveniencebased**

It should read:

**non-randomized, convenience-based**

On pages 4 and 5, where it reads:

***P*value**

It should read:

***P*-value**

On pages 4 and 5, where it reads:

**breedlevel**

It should read:

**breed-level**

On Table 1, page 5, where it reads:

**Genotypepositive**

It should read:

**Genotype-positive**

On page 6, where it reads:

**locusspecific**

It should read:

**locus-specific**

On page 6, where it reads:

**allelefrequency**

It should read:

**allele-frequency**

On page 6, where it reads:

**resourcelimited**

It should read:

**resource-limited**

On page 6, where it reads:

**fieldadapted**

It should read:

**field-adapted**

The authors apologize for the errors.

